# Kaiso Protein Expression Correlates with Overall Survival in TNBC Patients

**DOI:** 10.3390/jcm12010370

**Published:** 2023-01-03

**Authors:** Artur Bocian, Piotr Kędzierawski, Janusz Kopczyński, Olga Wabik, Anna Wawruszak, Michał Kiełbus, Paulina Miziak, Andrzej Stepulak

**Affiliations:** 1 Oncological Surgery Clinic, The Holycross Cancer Centre, 25-734 Kielce, Poland; 2 Collegium Medicum, Jan Kochanowski University, 25-317 Kielce, Poland; 3 Radiotherapy Department, The Holycross Cancer Centre, 25-734 Kielce, Poland; 4 Pathology Department, The Holycross Cancer Centre, 25-734 Kielce, Poland; 5 Department of Biochemistry and Molecular Biology, Medical University of Lublin, 20-093 Lublin, Poland; 6 Department of Experimental Hematooncology, Medical University of Lublin, 20-093 Lublin, Poland

**Keywords:** breast cancer, TNBC, Kaiso, TCGA, immunohistochemistry

## Abstract

Triple-negative breast cancers (TNBCs) are histologically heterogenic invasive carcinomas of no specific type that lack distinctive histological characteristics. The prognosis for women with TNBC is poor. Regardless of the applied treatments, recurrences and deaths are observed 3–5 years after the diagnosis. Thus, new diagnostic markers and targets for personalized treatment are needed. The subject of our study—the Kaiso transcription factor has been found to correlate with the invasion and progression of breast cancer. The publicly available TCGA breast cancer cohort containing Illumina HiSeq RNAseq and clinical data was explored in the study. Additionally, Kaiso protein expression was assessed in formalin-fixed and paraffin-embedded tissue archive specimens using the tissue microarray technique. In this retrospective study, Kaiso protein expression (nuclear localization) was compared with several clinical factors in the cohort of 103 patients with TNBC with long follow-up time. In univariate and multivariate analysis, high Kaiso protein but not mRNA expression was correlated with better overall survival and disease-free survival, as well as with premenopausal age. The use of radiotherapy was correlated with better disease-free survival (DFS) and overall survival (OS). However, given the heterogeneity of TNBC and context-dependent molecular diversity of Kaiso signaling in cancer progression, these results must be taken with caution and require further studies.

## 1. Introduction

Breast cancer remains the most frequently diagnosed female cancer and the cause of many cancer deaths, despite screening and improvements in adjuvant treatment. Breast cancer (BC) is a clinically heterogeneous disease encompassing about 15 different types of carcinomas, sub-classified according to their estrogen receptor (ER), progesterone receptor (PR), and human epidermal growth factor receptor 2 (HER2) status [1]. For the majority of patients, targeted therapies against these targets are available. Such treatment options are absent for patients diagnosed with tumors lacking ER, PR, and HER2, referred to as triple-negative breast cancers (TNBC). The prognosis for women with TNBC is worse in comparison to women with luminal types of breast cancer. In patients with TNBC, regardless of the applied treatments, recurrences and deaths are observed during 3–5 years after the diagnosis [2,3,4,5]. Heterogeneity of these tumors and different routes of metastatic spread may explain the higher recurrence and mortality rates of TNBC patients [6,7]. The analysis from a large number of TNBCs identified six stable and biologically different clusters of TNBC exhibiting unique gene expression patterns and gene ontologies [8], suggesting a differential response to selected chemotherapeutics for patients bearing a specific molecular subtype of TNBC [9]. Thereby, there is an urgent need for the identification of biomarkers that could improve prognosis or predict the therapeutic outcomes of TNBC patients.

Kaiso is a BTB/POZ (Broad complex, Tramtrack and Bric à brac/POxvirus and Zinc finger, hereafter POZ) protein–protein interaction domain and a carboxy-terminal zinc finger (ZF) domain transcription factor. Depending on the cell type and tissue context, post-translational modifications, and binding partners, Kaiso functions as a transcriptional repressor or activator. Kaiso negatively regulates the expression of many genes in a methylation-dependent manner or binds to unmethylated DNA sequences termed Kaiso Binding Site (KBS), which activates Kaiso transcriptional activity [10]. On the protein level, covalent linkage of small ubiquitin-like modifier (SUMO) polypeptides to the Kaiso protein forms a molecular switch—SUMOylated Kaiso acts as an activator, whereas de-SUMOylated Kaiso acts as a repressor [11]. Similarly, Kaiso seems to activate or repress pro-apoptotic genes by interaction with wild-type or mutated p53, respectively [10].

Kaiso is expressed in multiple cell types [12] and human cancers, including colon [13], lung [14], prostate [15], and breast tumors. High Kaiso expression (nuclear localization) in BC was correlated with estrogen receptor-α negativity, and was present in the HER2-driven and basal/TNBCs [16]. The expression of Kaiso in BC was evaluated mainly in patient cohorts divided into invasive lobular carcinoma (ILC) and infiltrating ductal carcinoma (IDC) (9,10). Rare are reports dealing with the clinical significance of Kaiso expression in TNBC patients only [17]. Thereby, in our retrospective study, we compared Kaiso protein expression with other clinical factors in the cohort of 103 patients with TNBC with a long follow-up time after treatment. In addition, we enriched our studies with Illumina HiSeq RNAseq data, to determine the correlation between *ZBTB33* transcript expression and overall survival (OS) and progression-free interval (PFI) in the TNBC cohort. We demonstrated that Kaiso protein but not *ZBTB33* transcript expression positively correlates with OS in TNBC patients.

## 2. Materials and Methods

### 2.1. Open Data Sources

The publicly available TCGA breast cancer cohort which contained Illumina HiSeq RNAseq and clinical data was explored. TCGA data were hosted by the NCI’s Genomic Data Commons (GDC) at https://portal.gdc.cancer.gov/ (accessed on 20 December 2022), whereas overall survival (OS) and progression-free interval (PFI) data were purchased from a previously published paper [18]. A total of 192 TNBC samples were identified according to the criteria described in the publication [19]. GC-content normalized RNAseq data were used for extracting *ZBTB33* expression for each sample. Survival 3.4-0 and Survminer 0.4.9 R packages were used for survival analysis [20,21]. The optimal cutpoint for *ZBTB33* was estimated using the maximally selected rank statistics from the maxstat R package. Statistical analysis and data visualization (Kaplan–Meier curves, Cox proportional hazards regression analysis) were performed using R 4.1.3 environment (R Foundation for Statistical Computing, San Francisco, CA, USA).

### 2.2. Patients

A total of 103 women with TNBC were enrolled into the study. All diagnostic, therapeutic, and follow-up procedures were conducted in one center—Holy Cross Cancer Center in Kielce (HCCK), Poland during 2011–2014. The mean age of the patients was 57.4 years (ranged from 22 to 89). There were 36 (35%) patients in pre-menopausal age and 67 (65%) in post-menopausal age. In most patients (79.6%), cancer was diagnosed in the I or II clinical stages. All patients were treated surgically. Chemotherapy was applied preoperatively in 27 patients, and postoperatively in 82 patients. Postoperative chemotherapy was followed by conformal radiotherapy in 72 patients (69.9%). Detailed patient characteristics and the types of treatment are depicted in Table 1.

The cancer tissues were derived from the archives of the Department of Pathology of the HCCK. The use of anonymous patient biological material for scientific purposes is part of the standard treatment contract with patients in Poland. In accordance with article 26 [3] of the Act on Patient’s Rights and Patient’s Ombudsman of 2008, November the 6th (i.e., Journal of Laws of 2016, item 186 as amended), medical records can be shared with universities or research institutes to be used for scientific purposes without revealing surnames or other data enabling identification of the person to which it relates. According to the judgment of the Supreme Court of Poland number VCSK 256/10, tissue sections from a human organism and histopathological specimen constitute medical records. Thereby, tissue sections and specimen can be shared with medical colleges according to the law under conditions other than patient’s consent. Ethical approval was not required.

### 2.3. Immunohistochemistry

Formalin-fixed and paraffin embedded tissue blocks of 103 TNBC patients were utilized to construct tissue microarray. First, 4 µm thick tissue sections were warmed up at 58 °C for 1 h, deparaffinized, and subjected to antigen retrieval in pH 9.0 EnVision FLEX Solution (Dako Omnis; 1:50) at 98 °C for 20 min, then rinsed in Wash Buffer (Dako Omnis) for 5 min. Endogenous peroxidase activity was blocked for 5 min in EnVision FLEX Peroxidase—Blocking Reagent (Dako Omnis) containing 3% hydrogen peroxide. After rinsing in Wash Buffer (Dako Omnis) for 5 min, tissue slides were incubated with HRP-conjugated mouse monoclonal Kaiso antibody (1:50; 6F8, clone sc-23871, Santa Cruz Biotechnology, Inc., Dallas, TX, USA) for 35 min in room temperature, followed by rinsing in Wash Buffer (Dako Omnis, Copenhagen, Denmark) for 5 min. Tissues were sequentially incubated in EnVision FLEX/ HRP (Dako Omnis) for 20 min, rinsed in Wash Buffer (Dako Omnis), incubated twice in DAB-Chromogen (Dako Omnis) for 5 min, and then rinsed in distilled water. Counterstaining was achieved by incubating tissues in Mayer’s hematoxylin (Dako Omnis) for 5 min, followed by rinsing in distilled water. Slides were then dehydrated in ascending alcohol dilutions, cleared with xylenes for 5–7 min and mounted using DPX (Dako Toluen-Free Mounting Medium, Dako, Glostrup, Denmark). The human urinary bladder tissue was used as the positive control and negative controls were obtained by excluding antibody. Briefly, cells were blindly scored by two pathologists with similar results. Individual specimens were scored for membranous, cytoplasmic, and nuclear staining for Kaiso and classified with respect to the intensity of immunostaining, with the percentage of cells determined at each staining intensity from 0 to +2. Respective staining intensity: negative (0; <5%), low (+1; >5–50%), and high (+2; >50%) are presented in Section 3.

### 2.4. Statistical Analysis

Descriptive statistics for continuous variables and frequencies for category variables were used for the characteristics of the study group. The Kruskal–Wallis and chi-square test was used to study the correlations between Kaiso expression and clinical or pathology-related factors. The results were evaluated in terms of overall survival (OS) and disease-free survival (DFS). The 1-year, 3-year, and 5-year survival rates were estimated using the Kaplan–Meier method. The influence of selected factors (age, clinical stage, postoperative chemotherapy, preoperative chemotherapy, radiotherapy, and Kaiso expression) on the patient prognosis was assessed with the Cox proportional hazards model (univariate and multivariate). All parameters which were statistically significant in the univariate analysis were estimated in the multivariate analysis. It was assumed that *p*-values below 0.05 mean statistical significance. Statistical analyses were performed using MedCalc Statistical Software version 19.1 (MedCalc Software bv, Ostend, Belgium; https://www.medcalc.org, accessed on 20 December 2022).

## 3. Results

**Figure 1:**
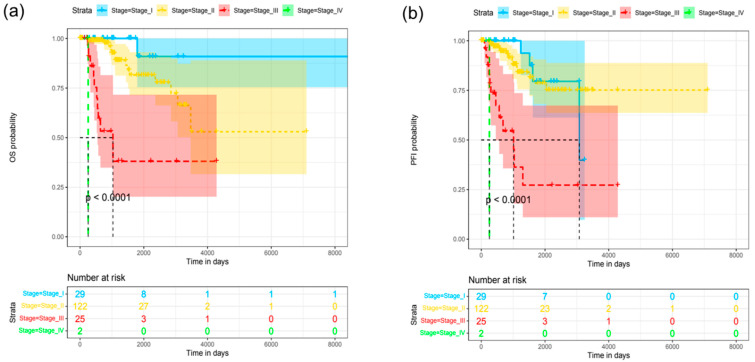
Survival probability of TCGA TNBCs patients sub-grouped by clinical stage. (**a**) Overall survival (OS), (**b**) progression-free interval (PFI).

**Figure 2:**
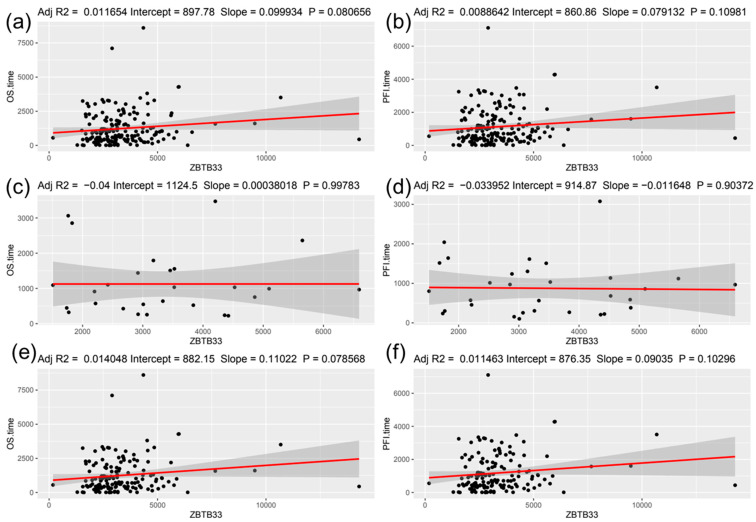
Scatter plots and linear regression lines of clinical data (OS and PFI) and *ZBTB33* expression in RNAseq TNBCs TCGA cohort. (**a**) Overall survival (OS) for all patients, (**b**) progression-free interval (PFI) for patients who died from any cause, (**c**) OS for only event patients, (**d**) PFI for patients for dead patients or having new tumor event, (**e**) OS for the censored patient group, (**f**) PFI for the censored patient group.

**Figure 3:**
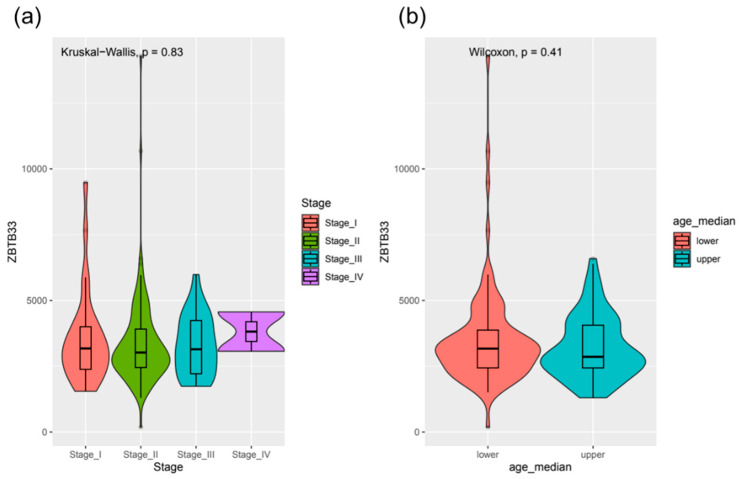
*ZBTB33* expression in RNAseq TNBCs TCGA cohort by: (**a**) clinical stage (Stage I–IV), (**b**) upper or lower than median age (median_age).

For evaluating the clinical importance of Kaiso for TNBC prognosis or progression, first we looked into transcriptomic data for 192 TCGA patients for which clinical data containing overall survival (OS) and progression-free interval (PFI) are publicly available. Validation of the dataset revealed that OS probability estimated using the Cox model was significant only for clinical Stage III (Hazard ratio (HR) 25.42, *p* = 0.002) and IV (HR 324.04, *p* < 0.001) TNBC patients (Figure 1a). Similarly, PFI probability was seen as significant for clinical Stage III (HR 7.52, *p* = 0.001) and IV (HR 34.15, *p* < 0.003) patients (Figure 1b). We did not see any significant relationship between OS or PFI time with the *ZBTB33* transcript level using linear regression (Figure 2a–f, *p* > 0.05). Moreover, *ZBTB33* expression did not show significant changes (*p* > 0.05) between patients grouped by clinical stage or median age at diagnosis (Figure 3a,b). According to that, we were unable to determine the optimal cut-off point of *ZBTB33* expression for estimating significant changes in OS or PFI probability using the Cox model in the analyzed dataset. 

**Figure 4:**
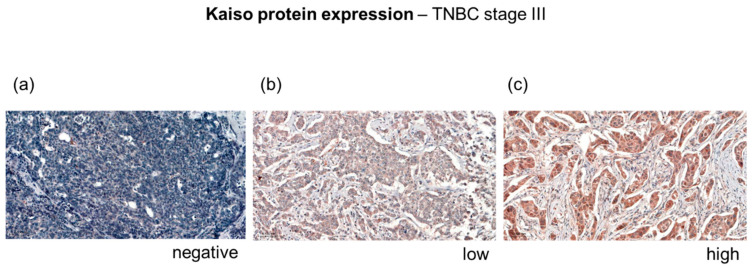
Kaiso immunostaining of TNBC tissues (III grade). IHC images at 40 × magnification show differential staining intensity pattern of Kaiso expression: (**a**) negative (0; <5%), (**b**) low (+1; >5–50%), and (**c**) high (+2; >50%).

**Figure 5:**
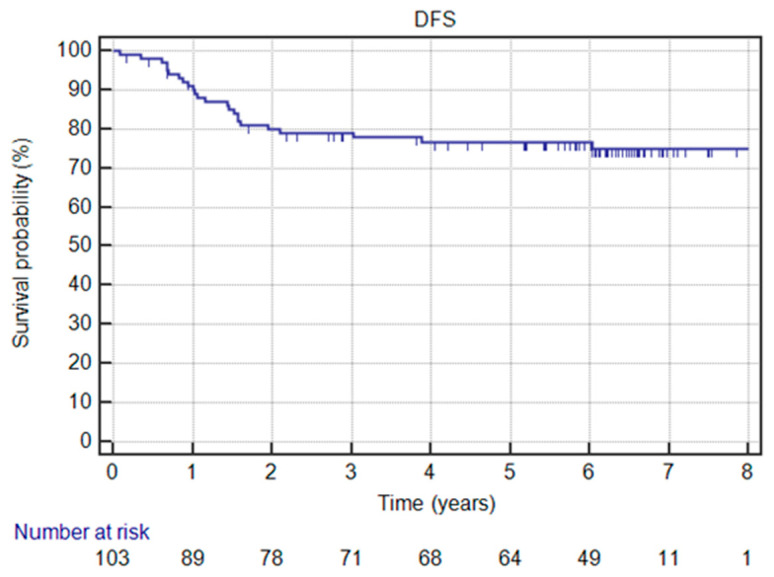
Disease-free survival (DFS) in the whole group.

**Figure 6:**
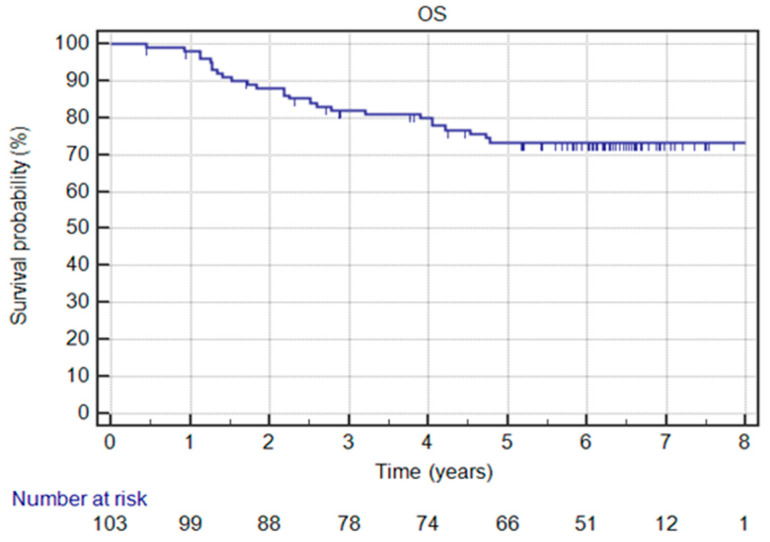
Overall survival (OS) in the whole group.

According to our protein expression studies (Figure 4), Kaiso no expression was found in 19 (18.4%) patients, low expression in 38 (36.9%) patients, whereas high ex-pression was detected in 46 (44.7%) patients (Table 2). In univariate and multivariate analysis, high Kaiso expression was correlated with better disease-free survival (Table 3 and Table 4) (Figure 5) and overall survival (Table 3 and Table 5) (Figure 6). From different clinical factors analyzed, only premenopausal age was statistically correlated with high expression of Kaiso. Age, clinical stage, histological tumor grading, as well as the number of patients with recurrence of cancer disease and the number of patients who died were not in association with Kaiso expression (Table 2).

From the clinical point of view, we noted 26 (25.2%) patient deaths and 24 (23.3%) recurrences of the cancer disease in the analyzed patients’ group during the observation time (2011–2019). Four patients are alive with dissemination after salvage treatment (one of them in breast, bones, brain, and mediastinal lymph nodes). Based on the analysis of the Kaplan–Meier estimator of the survival function, we found that the probability of 5-year DFS and OS were 76.7% and 79.9%, respectively (Figure 5 and Figure 6). Clinical factors significantly correlating with better OS were younger age and earlier clinical-stage (Table 5). The use of radiotherapy had an influence on better DFS and OS [22] (Table 4 and Table 5).

## 4. Discussion

TNBCs are classified histologically as invasive mammary carcinomas of no specific type that lack distinctive histological characteristics [23]. Despite the identification of TNBC molecular subtypes [8], a correlation between the current clinical therapeutic strategies and the histological and molecular complexity of TNBC has not been found [23].

In the past decades, comprehensive research efforts have been put into finding new predictive molecular markers for the development of diagnostic and therapeutic methods in breast cancer. In this regard, several antigens, e.g., BRCA1, BRCA2, BR 27.29, CA 15-3, CA 27.29, CEA, c-myc, HER-2/neu, MUC-1, or p53 have been reported for monitoring breast cancer progression [24]. The subject of our study—Kaiso transcription factor—did not fall into the molecular markers list for TNBC patients yet. However, it has demonstrated alterations in Kaiso expression in this subtype of breast cancer. To address this topic, we performed a comprehensive evaluation of mRNA and protein Kaiso expression in publicly available databases and using tissue microarray techniques in TNBC samples, respectively.

According to GDC Data Portal OS and PFI in the TCGA, TNBCs cohorts were significantly decreased for advanced clinical stages, which corresponds to previously published results [19]. However, we did not observe any significant relationship between OS or PFI time with the *ZBTB33* transcript level using linear regression. Moreover, *ZBTB33* mRNA expression did not show significant changes between patients grouped by clinical stage or median age at diagnosis. Despite the fact that the *ZBTB33* mRNA abundance was reported as predictive of poor overall breast cancer survival in publicly available Metabric cohorts of 555 patients who underwent surgery for their primary breast cancer tumor, we are not able to easily compare this result to only TNBCs patients analyzed in our study [25]. Interestingly, *ZBTB33* mRNA levels do not correlate with either nuclear or cytoplasmic levels of Kaiso [25,26]. This evidence suggests that the clinical relevance of *ZBTB33* mRNA levels for TNBC patients is severely limited. Despite the lack of correlation between *ZBTB33* transcript level and OS and PFI, we observed a positive correlation between Kaiso protein expression and these variables. To the best of our knowledge, this is the first multivariate analysis that demonstrated a statistically positive correlation between Kaiso protein expression and OS and DFS of TNBC patients. In contrast to our results, another group’s findings reveal that high Kaiso expression correlates with invasion, lymph node metastases, and the reduced overall survival of patients with invasive ductal carcinoma (IDC) [27] or high-grade BC [16]. In this context, the results of our studies are, to some extent, contradictory to these reports. The reasons for the observed discrepancy could be multifactorial. From the clinical point of view, in contrast to the majority of published data [28,29], our homogenous group of patients (TNBC only, Polish population, one diagnostic, and treatment center) had recognized cancer in I and II stages in almost 80% of cases, and display G2/G3 histological tumor grading (97%), which differs our cohort from that of other studies and potentially influences results. Interpatient- and tumor-related heterogeneity could play a role as well, e.g., in our studies, Ki-67 proliferative marker ranged between 1% and 95% for particular tumors. Applied treatment procedures—surgical removal of low advanced tumors in all analyzed patients could influence OS and DFS, as demonstrated for radiotherapy.

On the other hand, Kaiso itself has been reported to play opposite molecular roles depending on cellular context, epigenetic changes—either DNA or protein modifications and p53 protein mutation status [10]. In vitro studies revealed a role of Kaiso in the proliferation and survival of TNBC cells, since Kaiso downregulation attenuates cell proliferation, whereas in vivo delays tumor onset in mice xenografted with the aggressive MDA-MB-231 breast cancer cells [30]. However, the lack of influence of tumor microenvironment in typical cell-culture-related studies does not reflect the conditions of tumor growth in human body. Moreover, several studies in other cancer types suggest a pro-oncogenic role for Kaiso [10,15,16], whereas others associate Kaiso with a tumor suppressive role [31,32], which makes the potential function of Kaiso in cancer progression even more complicated.

Kaiso interferes with several signaling pathways. TGFβ (Tumor necrosis factor β) signaling induced Kaiso expression in TNBC cells at both the transcript and protein levels [33]. TGFβ signaling plays a paradoxical role in breast cancer; in early-stage breast cancer, TGFβ acts as a tumor suppressor by inhibiting cell proliferation and inducing apoptosis, but in advanced stages, TGFβ promotes progression and metastasis partly through induction of EMT (epithelial-to-mesenchymal transition) [34]. Since, in our studies, the patients had recognized cancer in I and II stages in almost 80% of cases, such diversity in molecular function of Kaiso and related molecular pathways could influence our results as well. These and other studies highlight paradoxical roles for Kaiso in apoptosis and indicate that even within the same tissue type, Kaiso’s role is highly complex and context-dependent. Thus, combining routine histological examinations with molecular analysis could be a balanced approach to assess and predict the invasiveness and clinical behavior of particular tumors, providing the link between the current clinical therapeutic guidelines and the molecular complexity of TNBC.

## 5. Conclusions

Kaiso is a multi-functional and bimodal transcriptional modulator, which can play both tumor promotor and tumor suppressive roles depending on the molecular context and type of neoplasm. Recent studies have found a correlation between altered Kaiso expression and the aggressiveness of cancer. In our studies, using publicly available databases and tissue microarray techniques we have demonstrated that Kaiso protein but not mRNA expression correlates with OS and DFS of TNBC patients. This evidence suggests that the clinical relevance of *ZBTB33* mRNA (in contrast to Kaiso protein expression) levels for TNBC patients is severely limited. However, given the heterogeneity of TNBC and context-dependent molecular diversity of Kaiso’s role in cancer progression, further molecular investigations are needed.

## Figures and Tables

**Table 1:** Characteristics of study group.

N	Factor	Total
1	Number of patients	103
2	Observation period (years)	
	mean (SD)	5.0 (2.1)
	median (Q1–Q3)	5.9 (3.4–6.6)
	min–max	0.5–8.1
3	Age	
	mean (SD)	57.0 (13.4)
	median (Q1–Q3)	57 (50–67)
	min–max	22–89
4	Age	
	≤57	55 (53.4%)
	>57	53 (46.6%)
5	Premenopausal	36 (35.0%)
	Postmenopausal	67 (65.0%)
6	Clinical stage	
	I	21 (20.4%)
	II	61 (59.2%)
	III	18 (17.5%)
	IV	3 (2.9%)
7	Histopathology	
	No special type	99 (96.1%)
	Other	4 (3.9%)
8	Grading	
	1	3 (2.9%)
	2	59 (57.3%)
	3	41 (39.8%)
9	Ki 67	
	mean (SD)	50.9 (24.4)
	median (Q1–Q3)	50 (30–70)
	min–max	1–95
10	Ki 67	
	≤50	59 (57.3%)
	>50	44 (42.7%)
11	Preoperative chemotherapy	
	Yes	27 (26.2%)
	No	76 (73.8%)
12	Postoperative chemotherapy	
	Yes	82 (79.6%)
	No	21 (20.4%)
13	Surgery	
	Breast conserving treatment (BCT)	26 (25.2%)
	Radical mastectomy (RM)	69 (67.0%)
	Simple mastectomy (SM)	3 (2.9%)
	Subcutaneous mastectomy (SSM)	5 (4.9%)
14	Axillary dissection	78 (75.7%)
	Sentinel node biopsy	25 (24.3%)
15	Radiotherapy	
	Yes	72 (69.9%)
	No	31 (30.1%)
16	Reccurence	
	No	79 (76.7%)
	Yes	24 (23.3%)
17	Death	
	No	83 (81.6%)
	Yes	20 (19.4%)
18	Kaiso Expression	
	0—absent	19 (18.4%)
	1—low	38 (36.9%)
	2 + 3—medium and high	46 (44.7%)

**Table 2:** Kaiso expression and clinical parameters.

Factor	Kaiso 0	Kaiso 1+	Kaiso 2+, 3+	*p*-Value
Number of patients—N (%)	19 (18.4%)	38 (36.9%)	46 (44.7%)	
Age				0.171
mean (SD)	62.1 (11.4)	56.7 (12.4)	55.2 (14.6)	
median (Q1–Q3)	63 (54–68)	58 (51–64)	5 (47–66)	
min–max	39–89	22–78	25–86	
Age				0.1361
≤57	7 (36.8%)	19 (50.0%)	29 (63.0%)	
>57	12 (63.2%)	19 (50.0%)	17 (37.0%)	0.0141
Premenopausal	17 (89.5%)	26 (68.4%)	24 (52.2%)	
Postmenopausal	2 (10.5%)	12 (31.6%)	22 (47.8%)	
Clinical stage				0.9468
I	4 (21.1%)	9 (23.7%)	8 (17.4%)	
II	10 (52.6%)	21 (55.3%)	30 (65.2%)	
III	4 (21.1%)	7 (18.4%)	7 (15.2%)	
IV	1 (5.3%)	1 (2.6%)	1 (2.2%)	
Grading				0.7923
1	1 (5.3%)	1 (2.6%)	1 (2.2%)	
2	9 (47.4%)	21 (55.3%)	29 (63.0%)	
3	9 (47.4%)	16 (42.1%)	16 (34.8%)	
Ki 67				0.097
mean (SD)	48.2 (25.8)	57.7 (23.7)	46.5 (23.5)	
median (Q1–Q3)	50 (23–68)	60 (50–80)	60 (50–80)	
min–max	5–90	1–95	5–90	
Ki 67				0.0656
≤50	10 (52.6%)	17 (44.7%)	32 (69.6%)	
>50	9 (47.4%)	21 (55.3%)	14 (30.4%)	
Recurrence				0.2403
No	12 (63.2%)	29 (76.3%)	38 (82.6%)	
Yes	7 (36.8%)	9 (23.7%)	8 (17.4%)	
Death				0.2573
No	13 (68.4%)	26 (68.4%)	38 (82.6%)	
Yes	6 (31.6%)	12 (31.6%)	8 (17.4%)	

**Table 3:** Disease-free and overall survival in the whole group.

Years of Observation	Survival Probability (Standard Deviation)	
	Disease-Free Survival (DFS)	Overall Survival (OS)
1	91.1 (2.8)	98.0 (1.4)
3	78.9 (4.1)	82.1 (3.8)
5	76.7 (4.3)	79.9 (4.0)
Number of events (%)	24 (23.3)	26 (25.2)
Number of censored (%)	79 (76.7)	77 (74.8)
Mean survival * (95% CI)	6.5 (5.9–7.0)	6.6 (6.1–7.1)

* HR—hazard ratio.

**Table 4:** Cox regression analysis for disease-free survival (DFS).

Variables	Univariate Analysis		Multivariate Analysis	
	HR (95% CI) *	*p*-Value	HR (95% CI)	*p*-Value
Age	1.04 (1.00–1.07)	0.0374	1.03 (1.00–1.07)	0.0390
Clinical stage	2.44 (1.28–4.65)	0.0066	1.76 (0.83–3.74)	0.1443
Postoperative chemotherapy	0.37 (0.16–0.87)	0.0228	0.68 (0.26–1.75)	0.4224
Preoperative chemotherapy	2.99 (1.34–6.71)	0.0075	2.61 (0.85–8.07)	0.0954
Radiotherapy	0.39 (0.18–0.89)	0.0234	0.21 (0.87–0.52)	0.0007
Kaiso expression	0.62 (0.37–1.04)	0.0721	0.47 (0.26–0.84)	0.0111

* HR—hazard ratio.

**Table 5:** Cox regression analysis for overall survival (OS).

Variables	Univariate Analysis		Multivariate Analysis	
	HR (95% CI)	*p*-Value	HR (95% CI)	*p*-Value
Age	1.06 (1.02–1.09)	0.0012	1.06 (1.02–1.09)	0.0008
Clinical stage	2.27 (1.33–3.87)	0.0027	2.20 (1.09–4.46)	0.0286
Postoperative chemotherapy	0.37 (0.16–0.83)	0.0155	0.78 (0.33–1.85)	0.5696
Preoperative chemotherapy	3.03 (1.40–6.57)	0.0049	2.84 (0.95–8.51)	0.0619
Radiotherapy	0.34 (0.16–0.73)	0.0056	0.17 (0.07–0.41)	0.0001
Kaiso expression	0.67 (0.41–1.10)	0.1159	0.47 (0.27–0.81)	0.0067

## Data Availability

All datasets generated for this study are included in the manuscript.
